# Folic acid inhibits 5‐methyltetrahydrofolate transport across the blood–cerebrospinal fluid barrier: Clinical biochemical data from two cases

**DOI:** 10.1002/jmd2.12321

**Published:** 2022-08-06

**Authors:** Tomoyuki Akiyama, Ichiro Kuki, Kiyohiro Kim, Naohiro Yamamoto, Yumi Yamada, Kazuya Igarashi, Tomohiko Ishihara, Yuya Hatano, Katsuhiro Kobayashi

**Affiliations:** ^1^ Department of Child Neurology Okayama University Hospital Okayama Japan; ^2^ Department of Child Neurology, Okayama University Graduate School of Medicine Dentistry and Pharmaceutical Sciences Okayama Japan; ^3^ Department of Pediatric Neurology Osaka City General Hospital Osaka Japan; ^4^ Department of Pediatric Neurology Hyogo Prefectural Amagasaki General Medical Center Hyogo Japan; ^5^ Department of Neurology National Hospital Organization Nishiniigata Chuo Hospital Niigata Japan; ^6^ Department of Neurology, Brain Research Institute Niigata University Niigata Japan

**Keywords:** 5‐formyltetrahydrofolic acid, cerebral folate deficiency, folate receptor 1, folinic acid, Kearns‐Sayre syndrome, methylenetetrahydrofolate reductase deficiency

## Abstract

**Objective:**

The use of folic acid (FA) has been discouraged in cerebral folate deficiency (CFD) because, theoretically, it could inhibit the transport of 5‐methyltetrahydrofolic acid (5MTHF) across the blood–cerebrospinal fluid (CSF) barrier. We present the clinical biochemical data of two cases with CFD to support this hypothesis.

**Methods:**

We measured CSF and serum 5MTHF concentrations in a patient with Kearns‐Sayre syndrome (KSS) and a patient homozygous for *MTHFR* C677T polymorphism before and during folate supplementation therapy. To evaluate these 5MTHF concentrations, we also analyzed CSF and serum samples in pediatric patients without folate supplementation.

**Results:**

Both patients had low CSF 5MTHF before treatment and high‐dose FA therapy did not normalize CSF 5MTHF. There was a dissociation between serum total folate and 5MTHF concentrations during FA therapy, which was considered to be due to the appearance of unmetabolized FA. The addition of folinic acid did not improve low CSF 5MTHF in the KSS patient and the cessation of FA resulted in the normalization of CSF 5MTHF. In the patient homozygous for *MTHFR* C677T, minimization of the FA dosage resulted in the normalization of CSF 5MTHF and an increased CSF‐to‐serum 5MTHF ratio.

**Conclusions:**

Our data suggest that excess supplementation of FA impaired 5MTHF transport across the blood–CSF barrier. In the treatment of CFD, supplementation of folinic acid or 5MTHF (in cases of impaired 5MTHF synthesis) is preferred over the use of FA. The reference values of CSF 5MTHF concentration based on 600 pediatric cases were also provided.

**SYNOPSIS**

Clinical biochemical data in our patients with cerebral folate deficiency suggest that excess supplementation of folic acid (FA) impairs 5‐methyltetrahydrofolic acid transport across the blood–CSF barrier and support the idea that high‐dose FA should not be used in this condition.

## 1 INTRODUCTION

**FIGURE 1:**
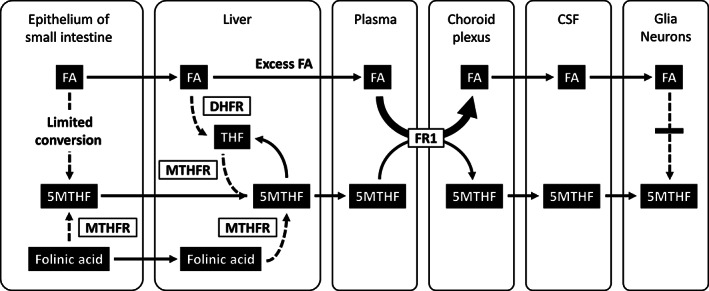
Transport of folate compounds from the intestine to the brain and competitive inhibition of 5MTHF transport by FA. Conversion of FA to 5MTHF is limited in the intestine and is mainly handled by DHFR in the liver, although its enzymatic activity is low in humans. In contrast, folinic acid is efficiently metabolized to 5MTHF in the intestine and liver. When an excess amount of FA is taken, it cannot be fully reduced by DHFR in the liver and unmetabolized FA appears in the plasma. Because FA has higher affinity to FR1 expressed at the choroid plexus than 5MTHF, it can act as a competitive inhibitor against 5MTHF transport from the plasma to the CSF. In addition, FA cannot be metabolized to 5MTHF efficiently in the brain with extremely low DHFR activity. Thus, excess FA intake may lead to a less effective supply of 5MTHF to the brain compared with that of folinic acid supplementation. Dashed arrows indicate more than one‐step enzymatic reactions. 5MTHF, 5‐methyltetrahydrofolic acid; CSF, cerebrospinal fluid; DHFR, dihydrofolate reductase; FA, folic acid; FR1, folate receptor 1; MTHFR, methylenetetrahydrofolate reductase; THF, tetrahydrofolate

Folate compounds (folates) act as one‐carbon donors in enzymatic reactions and play a critical role in purine and thymidylate biosynthesis and deoxyribonucleic acid (DNA) methylation.^1^ Folic acid (FA), commonly used for food fortification and supplements, is not biologically active and must be reduced by dihydrofolate reductase (DHFR) in the liver to be an active co‐factor (Figure 1). In the physiological condition, 5‐methyltetrahydrofolic acid (5MTHF) is the transport form of folate that enters the brain,^1^ and this is the main form of folate in the serum and cerebrospinal fluid (CSF).^1^, ^2^, ^3^

Cerebral folate deficiency (CFD) is a medical condition in which the 5MTHF in the brain is depleted. Insufficient 5MTHF in the brain can cause developmental delay, developmental deterioration, epileptic seizures, psychiatric symptoms, and leukoencephalopathy. CFD is caused either by impaired transport of 5MTHF across the blood–CSF barrier or by peripheral 5MTHF deficiency. Impaired 5MTHF transport to the CSF has been reported in several disorders, such as folate receptor 1 (FR1) deficiency caused by *FOLR1* gene abnormalities^4^ and Kearns‐Sayre syndrome (KSS).^5^ Peripheral 5MTHF deficiency is caused by nutritional folate deficiency, reduced folate absorption from the intestine, and inborn errors of folate metabolism affecting 5MTHF biosynthesis including methylenetetrahydrofolate reductase (MTHFR) deficiency.

In terms of treatment, folinic acid (5‐formyltetrahydrofolic acid) has been preferentially used to increase CSF 5MTHF in CFD caused by impaired 5MTHF transport to the CSF.^4^, ^6^, ^7^, ^8^ The use of FA in this condition has been strongly discouraged because it might exacerbate the CSF 5MTHF deficiency through the competitive inhibition of 5MTHF transport.^1^, ^9^, ^10^ The use of FA has also been discouraged for MTHFR deficiency for a similar reason^11^ and it is not recommended in the current guidelines.^12^ Although this presumed mechanism sounds reasonable, clinical biochemical data supporting this idea has not been well documented, because of rarity to perform lumbar puncture in patients on high‐dose FA therapy.

In this study, we aimed to present two clinical cases where high‐dose FA therapy was considered to impair 5MTHF transport across the blood–CSF barrier. We also provided updated reference values of CSF 5MTHF concentration at our laboratory based on 600 pediatric cases.

## 2 CASE REPORTS

### 2.1 Patients

We included a pediatric patient with KSS who was treated with FA and subsequently with folinic acid at Osaka City General Hospital, and an adult patient homozygous for *MTHFR* C677T polymorphism (rs1801133, NM_005957.5:c.665C>T, NP_005948.3:p.Ala222Val) who was treated with FA at National Hospital Organization Nishiniigata Chuo Hospital. Both patients underwent lumbar puncture to investigate CSF folate status before and during treatment. This study was approved by the ethics committee of Okayama University Hospital (Approval #1604‐009) and carried out in accordance with the Declaration of Helsinki. Written informed consent was obtained from the patients, their guardians, or adult family members before the procedure.

### 2.2 The KSS patient

This female pediatric patient presented with complete atrioventricular block, external ophthalmoplegia, and retinitis pigmentosa. The diagnosis of KSS was made based on elevated CSF protein and lactate, characteristic abnormalities on brain magnetic resonance imaging (MRI), muscle pathology findings compatible with mitochondrial disease, and a single large deletion of mitochondrial DNA in the muscle tissue. She started treatment with l‐carnitine, coenzyme Q10, and FA.

**FIGURE 2:**
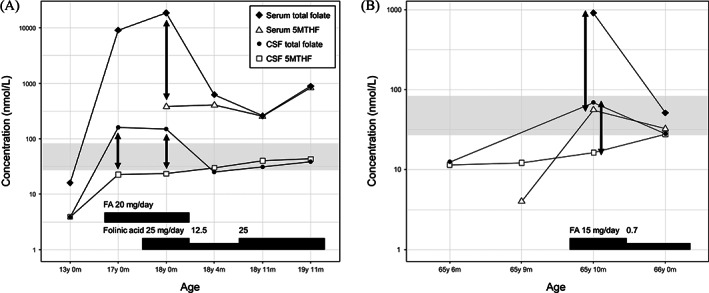
Concentrations of 5MTHF and total folate in serum and CSF samples in the patient with Kearns‐Sayre syndrome (KSS) and the patient homozygous for *MTHFR* C677T polymorphism. (A) Patient with KSS. (B) Patient homozygous for *MTHFR* C677T polymorphism. Complete cessation or dosage minimization of FA resulted in the normalization of CSF 5‐MTHF concentration. The dissociation between 5MTHF and total folate concentrations during FA therapy, which suggests the accumulation of unmetabolized FA, is demonstrated by vertical arrows. CSF 5MTHF reference value for 6–17 years old is indicated by the gray shaded area. 5MTHF, 5‐methyltetrahydrofolic acid; CSF, cerebrospinal fluid; FA, folic acid

The details of 5MTHF assay and reference values of CSF 5MTHF concentration at our laboratory based on 600 pediatric cases are described in Supplementary Material. The concentrations of 5MTHF and total folate in serum and CSF in this KSS patient are summarized in Figure 2A and Table S2. The patient demonstrated an extremely low concentration of CSF 5MTHF (3.9 nmol/L) before FA supplementation. The CSF 5MTHF‐to‐serum total folate ratio was also low (3.9/16.0 = 0.24, reference: 1.8–4.3^13^; 1.1–4.6, our data described in Supplementary Material), indicating reduced 5MTHF transport across the blood–CSF barrier. Supplementation of FA 20 mg/day elevated the CSF 5MTHF level but failed to achieve a normal value (26.8–83.2 nmol/L, our data described in Supplementary Material) despite the extremely high concentration (9062 nmol/L) of total folate in the serum. The addition of folinic acid 25 mg/day did not help increase CSF 5MTHF at all. Discontinuation of FA eventually succeeded in normalizing CSF 5MTHF, even with a lower dose (12.5 mg/day) of folinic acid. During FA therapy, there were significant dissociations between serum 5MTHF and total folate and between CSF 5MTHF and total folate, as demonstrated by the vertical arrows in Figure 2A.

### 2.3 The patient homozygous for MTHFR C677T polymorphism

This male adult patient presented with a sudden onset of impaired consciousness, generalized convulsive seizures, and cognitive decline. Brain MRI revealed T2‐hyperintense white matter lesions in the bilateral parietal and occipital lobes and the splenium of the corpus callosum. Blood tests revealed low serum total folate (2.9 nmol/L, reference value: ≥9.0 nmol/L), elevated plasma total homocysteine (71.6 μmol/L, reference value: 7.0–17.8 μmol/L), normal plasma methionine, and normal serum vitamin B12. A genetic test revealed that this patient was homozygous for *MTHFR* C677T polymorphism, but no other variants were found. He started treatment with vitamins B1, B6, B12, and FA.

Figure 2B and Table S3 summarize the concentrations of 5MTHF and total folate in serum and CSF samples. Reference values for CSF 5MTHF at 6–17 years old were applied because 5‐MTHF data of adults were not available at our laboratory. This patient had low concentrations (11.5 nmol/L and 12.2 nmol/L) of CSF 5MTHF before treatment. This was considered to be caused by low serum 5MTHF (4.0 nmol/L), not by impaired 5MTHF transport based on the normal CSF‐to‐serum 5MTHF ratio (12.2/4.0 = 3.05). FA therapy at 15 mg/day led to a minimal increase in CSF 5MTHF despite the high concentration of total folate (910.7 nmol/L) in the serum and moderate elevation of serum 5MTHF (55.7 nmol/L). At this point, the CSF‐to‐serum 5MTHF ratio was low (0.29) and there were significant dissociations between serum 5MTHF and total folate and between CSF 5MTHF and total folate, as indicated by the vertical arrows in Figure 2B. Reduction of FA dosage to 0.7 mg/day paradoxically increased CSF 5MTHF to the lower limit of normal level (27.7 nmol/L, reference value: 26.8–83.2 nmol/L, our data of 6–17 years old described in Supplementary Material) with a moderate increase in the CSF‐to‐serum 5MTHF ratio (27.7/32.6 = 0.85).

## 3 DISCUSSION

We presented clinical biochemical data demonstrating the inhibition of 5MTHF transport at the blood–CSF barrier by high‐dose FA and provided support for the idea that the use of FA should be avoided in CFD.^1^, ^9^, ^10^, ^11^ High‐dose FA increased the CSF 5‐MTHF concentration to some degree, which was likely associated with elevated serum 5‐MTHF, but normal levels were not reached in our patients. The lower efficacy of FA for 5MTHF supply to the CSF compared to folinic acid is mainly explained by the presence of excess FA in the blood and its antagonism against 5MTHF transport at the choroid plexus expressing FR1 (Figure 1). Low DHFR activity in the human liver^14^ leads to the appearance of unmetabolized FA in the blood upon high FA intake.^15^, ^16^, ^17^ The dissociation between 5MTHF and total folate concentrations during FA therapy in the KSS patient was likely due to the high amount of unmetabolized FA, although we were unable to measure unmetabolized FA directly. This hypothesis can be supported well by the absent or minimal dissociations after a complete switch to folinic acid therapy. The dissociation between 5MTHF and total folate concentrations during FA therapy in the patient homozygous for *MTHFR* C677T is likely explained by the accumulation of unmetabolized FA and also possibly by the elevation of reduced folate compounds other than 5MTHF due to the partial defect in MTHFR enzymatic activity.

5MTHF is considered to enter the brain via the blood–CSF barrier and the blood–brain barrier.^18^ CSF is considered to be the main source of 5MTHF supply to the brain because of its higher 5MTHF concentration than plasma.^19^ FR1 plays a main role in 5MTHF transport across the blood–CSF barrier at the choroid plexus at physiological serum 5MTHF concentrations.^1^, ^4^ This is supported by previous reports that FR1 deficiency resulted in CFD with extremely low CSF 5MTHF concentrations.^4^, ^8^, ^10^, ^20^, ^21^, ^22^, ^23^ Experiments in vitro demonstrated that FR1 has an approximately tenfold higher affinity to FA (*K*_*d*_ = 0.1 to 1 nmol/L) compared to 5MTHF (*K*_*d*_ = 1 to 10 nmol/L)^24^, ^25^, ^26^, ^27^, ^28^ and that FA competitively inhibits the binding of 5MTHF to FR1.^25^ This study demonstrated that serum 5MTHF concentration was only 2%–6% of serum total folate during high‐dose FA therapy, which suggests a considerable accumulation of unmetabolized FA that affects 5MTHF transport at the choroid plexus.

We also demonstrated that CSF 5MTHF concentration was approximately 15%–24% of CSF total folate during high‐dose FA therapy, suggesting the presence of unmetabolized FA in the CSF as well. The FA transported into the CSF instead of 5MTHF is biologically inactive and cannot be efficiently metabolized to 5MTHF in the central nervous system because of extremely low DHFR activity in the brain^29^ (Figure 1). In addition, FA was reported to inhibit MTHFR, causing pseudo‐MTHFR deficiency syndrome.^30^, ^31^ Conversely, folinic acid, a reduced form of folate, is rapidly metabolized to 5MTHF in the epithelium of the intestine.^32^ Therefore, folinic acid therapy results in a more efficient supply of 5MTHF to the brain in impaired 5MTHF transport across the blood–CSF barrier. With regard to patients with severe MTHFR deficiency, only betaine has shown effectiveness and is recommended by the guidelines.^12^ Although folate supplementation may be considered, FA and folinic acid do not elevate CSF 5MTHF but 5MTHF supplementation does.^33^ In partial MTHFR deficiency as in homozygous C677T polymorphism, FA supplementation may be permitted but dosage should be minimized to avoid the accumulation of unmetabolized FA. Folinic acid or 5MTHF supplementation, particularly the latter which can bypass MTHFR, is considered to be a simple and better choice.

There are some limitations to this study. Our speculation is based on only two patients. We were unable to measure FA directly, because it does not emit strong fluorescence. The difference between total folate and 5MTHF concentrations in our study may be explained by folate compounds other than FA, such as tetrahydrofolate, apart from methodological difference. Future studies that use liquid chromatography‐mass spectrometry to measure several folate compounds simultaneously will provide more information regarding folate status.^34^

## 4 CONCLUSION

We presented two clinical cases suggesting that the excess supplementation of FA impaired 5MTHF transport across the blood–CSF barrier. In the treatment of CFD, supplementation of folinic acid or 5MTHF (in cases with impaired 5MTHF synthesis) is preferred over FA, which may complicate 5MTHF transport to the CSF (the predominant 5MTHF source for the brain). The reference values of CSF 5MTHF concentration based on 600 pediatric cases were also provided.

## AUTHOR CONTRIBUTIONS

Tomoyuki Akiyama: conception and design, analysis and interpretation of data, drafting the article, guarantor. Ichiro Kuki, Kiyohiro Kim, Naohiro Yamamoto, Yumi Yamada, Kazuya Igarashi, Tomohiko Ishihara, Yuya Hatano, Katsuhiro Kobayashi: analysis and interpretation of data, revising the article for important intellectual content.

## CONFLICT OF INTEREST

The authors declare that they have no conflicts of interest.

## ETHICS STATEMENT

All procedures were conducted in accordance with the ethical standards of the responsible committee on human experimentation (institutional and national) and with the Declaration of Helsinki.

## INFORMED CONSENT

Informed consent was obtained from all patients for being included in the study.

## Supporting information


**Supplementary Figure S1** Chromatograms of serum 5MTHF assay using high‐performance liquid chromatography with fluorescence detection. (A) Standard solution of 5MTHF (256 nmol/L). 5MTHF elutes at approximately 2.3 min. (B) Serum sample from a 2‐month‐old patient (5MTHF: 54.3 nmol/L); 5MTHF, 5‐methyltetrahydrofolic acid
**Supplementary Figure S2** Concentration of 5MTHF in CSF samples from pediatric neurological patients. (A) CSF 5MTHF concentration vs. age. CSF 5MTHF declines with age. Quantile regression was performed using the formula, CSF 5MTHF = a log(age in month + 1) + b. The solid curve indicates the 50th percentile curve and the dashed curves indicate the 2.5 percentile and 97.5 percentile curves. (B) Boxplots of CSF 5MTHF concentration vs. four age groups. CSF 5MTHF concentrations in these groups are significantly different (*p* < 0.0001) from each other. 5MTHF, 5‐methyltetrahydrofolic acid; CSF, cerebrospinal fluid
**Supplementary Figure S3** Concentrations of 5MTHF and total folate in serum and CSF samples from pediatric neurological patients without folate supplementation or signs of peripheral folate deficiency. 5MTHF, 5‐methyltetrahydrofolic acid; CSF, cerebrospinal fluid
**Supplementary Table S1** Concentrations of 5MTHF and total folate in serum and CSF samples from pediatric neurological patients without folate supplementation or signs of peripheral folate deficiency
**Supplementary Table S2** Concentrations of 5MTHF and total folate in serum and CSF samples in the patient with Kearns‐Sayre syndrome
**Supplementary Table S3** Concentrations of 5MTHF and total folate in serum and CSF samples in the patient homozygous for *MTHFR* C677T polymorphism

## Data Availability

Data archiving is not mandated but data will be made available on reasonable request.
