# Supplementary material for: Folic acid inhibits 5‐methyltetrahydrofolate transport across the blood–cerebrospinal fluid barrier: Clinical biochemical data from two cases
Source: JIMD Rep. 2022 Aug 6;63(6):529–35. doi: 10.1002/jmd2.12321 (PMC9626660; doi:10.1002/jmd2.12321)
Supplement: Supplementary file 1 — Supplementary Figure S1 Chromatograms of serum 5MTHF assay using high‐performance liquid chromatography with fluorescence detection. (A) Standard solution of 5MTHF (256 nmol/L). 5MTHF elutes at approximately 2.3 min. (B) Serum sample from a 2‐month‐old patient (5MTHF: 54.3 nmol/L); 5MTHF, 5‐methyltetrahydrofolic acid Supplementary Figure S2 Concentration of 5MTHF in CSF samples from pediatric neurological patients. (A) CSF 5MTHF concentration vs. age. CSF 5MTHF declines with age. Quantile regression was performed using the formula, CSF 5MTHF = a log(age in month + 1) + b. The solid curve indicates the 50th percentile curve and the dashed curves indicate the 2.5 percentile and 97.5 percentile curves. (B) Boxplots of CSF 5MTHF concentration vs. four age groups. CSF 5MTHF concentrations in these groups are significantly different (p < 0.0001) from each other. 5MTHF, 5‐methyltetrahydrofolic acid; CSF, cerebrospinal fluid Supplementary Figure S3 Concentrations of 5MTHF and total folate in serum and CSF samples from pediatric neurological patients without folate supplementation or signs of peripheral folate deficiency. 5MTHF, 5‐methyltetrahydrofolic acid; CSF, cerebrospinal fluid Supplementary Table S1 Concentrations of 5MTHF and total folate in serum and CSF samples from pediatric neurological patients without folate supplementation or signs of peripheral folate deficiency Supplementary Table S2 Concentrations of 5MTHF and total folate in serum and CSF samples in the patient with Kearns‐Sayre syndrome Supplementary Table S3 Concentrations of 5MTHF and total folate in serum and CSF samples in the patient homozygous for MTHFR C677T polymorphism [file JMD2-63-529-s001.docx]

## Supplementary Material

## 1. Methods for 5-methyltetrahydrofolic acid (5MTHF) assay

### 1.1. Subjects to evaluate cerebrospinal fluid (CSF) and serum 5-methyltetrahydrofolic acid (5MTHF) concentrations

To update the reference values of CSF 5MTHF concentration we previously reported ^1^, we included pediatric patients with various neurological disorders who had a diagnostic work-up at Okayama University Hospital and other medical institutions. Those with pharmacological folate supplementation, known metabolic or genetic disorders affecting folate metabolism, or hemolyzed samples were excluded.

To evaluate the results of the 5MTHF assay for paired serum and CSF samples from patients with KSS and homozygous *MTHFR* C677T, we included pediatric patients with various neurological disorders who had had a diagnostic work-up at Okayama University Hospital. These patients were not on pharmacological folate supplementation and did not have signs of peripheral folate deficiency, including megaloblastic anemia or elevated plasma total homocysteine.

This study was approved by the ethics committee of Okayama University Hospital (Approval #1604-009) and carried out in accordance with the Declaration of Helsinki. Written informed consent was obtained from the patients, their guardians, or adult family members before the procedure.

### 1.2. Sample collection protocol

The lumbar puncture was performed after 4 to 6 hours of fasting. Paired serum samples were obtained immediately before the lumbar puncture. Serum and CSF samples were protected from light and frozen within 1 hour below −70°C. Samples collected outside Okayama University Hospital were shipped on dry ice to our laboratory. All samples were stored at −80°C until analysis.

### 1.3. Assay of 5MTHF

CSF 5MTHF was measured as per our previous report ^1^ with a slight modification described later. For serum 5MTHF assay, the samples were preprocessed by solid-phase extraction. Serum samples, 5MTHF calibrator (256 nmol/L), and quality control sample (64 nmol/L) (200 µL each) were diluted twofold with 4% phosphoric acid to release protein-bound 5MTHF. The Oasis HLB 1mL (Waters Japan, Tokyo, Japan) cartridges were preconditioned using 1 mL of acetonitrile followed by 1 mL of water, and then 360 µL of the diluted samples were loaded. After washing with 1 mL of the mobile phase A described below, 5MTHF was eluted by 180 µL of a mixture of the mobile phase A and acetonitrile (75:25, v/v). The eluted samples were diluted twofold by the diluent solution (water containing 1 g/L ascorbic acid). Finally, all processed samples were filtered through 0.45-µm Millex LH filters (Merck Millipore) and subjected to high-performance liquid chromatography (HPLC). The isocratic HPLC used the same mobile phases A and B as the previous study [#1864 Akiyama M, 2016]. The mobile phase A was made by dissolving 30 mmol (3.600 g) of sodium dihydrogenphosphate in 1 L of water and adjusting the pH to ≈2.3 with 1.8 mL of phosphoric acid. The mobile phase B was 100% acetonitrile. We slightly changed the ratio of mobile phase from A:B = 97:3 to A:B = 96.3:3.7 for better separation of the 5MTHF peak from neighboring peaks (particularly with serum samples).

### 1.4. Assay of total folate

We measured total folate concentrations in the serum and CSF using an automated analyzer with chemiluminescent enzyme immunoassay (UniCel DxI 800, Beckman Coulter, Brea, CA, USA).

### 1.5. Statistical analysis

Statistical analysis was conducted by R 4.1.2 (https://www.r-project.org/). Pearson’s correlation coefficient (r) and Spearman’s rank correlation coefficient (ρ) were calculated. Multiple comparison test was conducted by the Tukey–Kramer method. A test result was considered statistically significant when the *p*-value was below 0.05.

## 2. Results of 5MTHF assay

### 2.1. Validity of the serum 5MTHF assay by HPLC

5MTHF eluted at approximately 2.3 min and was well separated from other peaks (Supplementary Figure 1). The limit of quantitation (signal-to-noise ratio ≥10) was estimated to be approximately 2 nmol/L. The intra-day coefficient of variation (CV) was 0.1% (n = 5) and the inter-day CV was 2.6% (n = 5) using quality control samples at 64 nmol/L. Recovery was 90.7 to 109.8% for spiking serum samples by 25.6 nmol/L (n = 5) and 88.8 to 103.1% for spiking by 102.4 nmol/L (n = 5).

*2.2 Concentration of CSF 5MTHF in pediatric patients*

Supplementary Figure 2 presents a scatterplot of CSF 5MTHF concentration versus age and boxplots of CSF 5MTHF concentration versus four age groups. CSF 5MTHF concentration declined with age (ρ = −0.7525, *p* < 0.0001) and there were statistically significant differences among the four age groups (*p* < 0.0001, Tukey–Kramer test). The reference value was determined using p2.5 to p97.5; 55.5 to 179.6 nmol/L (median: 117.0 nmol/L) at 0 to 5 months old (n = 159), 45.8 to 127.6 nmol/L (median: 84.9 nmol/L) at 6 months to 1 year old (n = 145), 36.1 to 101.8 nmol/L (median: 66.7 nmol/L) at 2 to 5 years old (n = 127), and 26.8 to 83.2 nmol/L (median: 52.4 nmol/L) at 6 to 17 years old (n = 169).

### 2.3. Concentrations of serum total folate and 5MTHF, and CSF 5MTHF in pediatric patients without signs of peripheral folate deficiency

Supplementary Figure 3 and Supplementary Table 1 summarized the concentrations of serum total folate and 5MTHF, CSF 5MTHF, and plasma total homocysteine in 18 pediatric patients with neurological disorders without signs of peripheral folate deficiency. Plasma total homocysteine was not elevated (4.1–10.8 µmol/L) in all patients. There was a strong linear correlation (r = 0.9556, *p* < 0.0001) between serum total folate and 5MTHF concentrations (Supplementary Figure 3A). Their difference (5MTHF – total folate) ranged from −13.0 to 15.2 nmol/L (median: −0.3 nmol/L), which corresponded to the difference of −32.8 to 36.2% (median: −1.2%). Both serum (ρ = −0.6677, *p* = 0.0032) and CSF (ρ = −0.7399, *p* = 0.0007) 5MTHF concentrations demonstrated an age-dependent reduction (Supplementary Figure 3B). CSF-to-serum 5MTHF ratio, an indicator of 5MTHF transport across the blood–CSF barrier, showed a strong negative correlation (ρ = −0.9154, *p* < 0.0001) with serum 5MTHF concentration (Supplementary Figure 3C). There was a weak positive correlation (ρ = 0.4737, *p* = 0.0489) between age and CSF-to-serum 5MTHF ratio (Supplementary Figure 3D). The CSF-to-serum 5MTHF ratio ranged from 1.0 to 5.8 (median: 2.3) and the CSF 5MTHF-to-serum total folate ratio ranged from 1.1 to 4.6 (median: 1.9).

## References

1. Akiyama M, Akiyama T, Kanamaru K, et al. Determination of CSF 5-methyltetrahydrofolate in children and its application for defects of folate transport and metabolism. *Clin Chim Acta*. 2016;460:120-125.

## Supplementary Figure 1

Chromatograms of serum 5MTHF assay using high-performance liquid chromatography with fluorescence detection

A: Standard solution of 5MTHF (256 nmol/L). 5MTHF elutes at approximately 2.3 min. B: Serum sample from a 2-month-old patient (5MTHF: 54.3 nmol/L).

5MTHF, 5-methyltetrahydrofolic acid


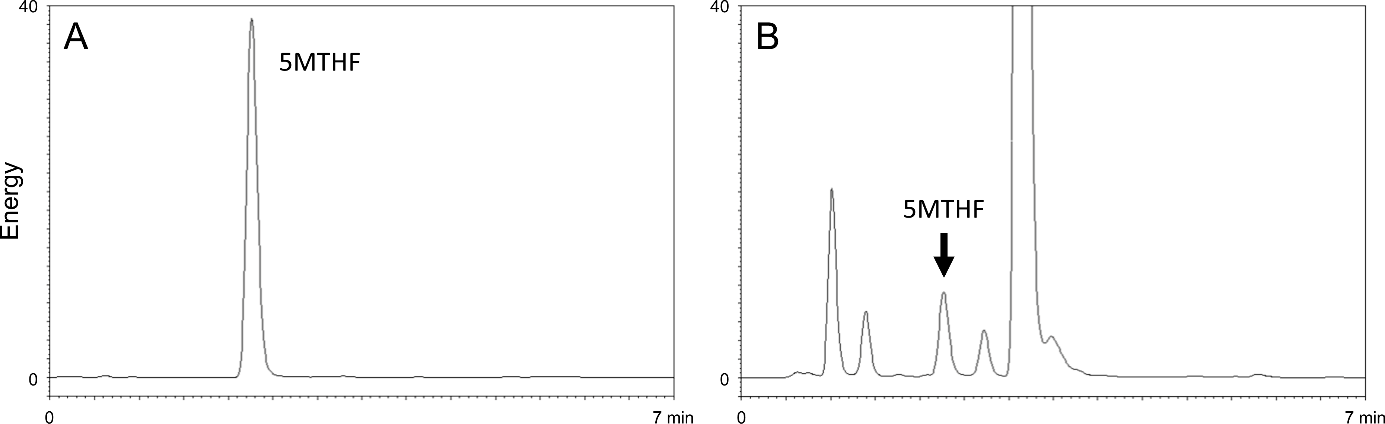


## Supplementary Figure 2

Concentration of 5MTHF in CSF samples from pediatric neurological patients.

A: CSF 5MTHF concentration vs. age. CSF 5MTHF declines with age. Quantile regression was performed using the formula, CSF 5MTHF = a log(age in month + 1) + b. The solid curve indicates the 50th percentile curve and the dashed curves indicate the 2.5 percentile and 97.5 percentile curves. B: Boxplots of CSF 5MTHF concentration vs. four age groups. CSF 5MTHF concentrations in these groups are significantly different (*p* < 0.0001) from each other.

5MTHF, 5-methyltetrahydrofolic acid; CSF, cerebrospinal fluid


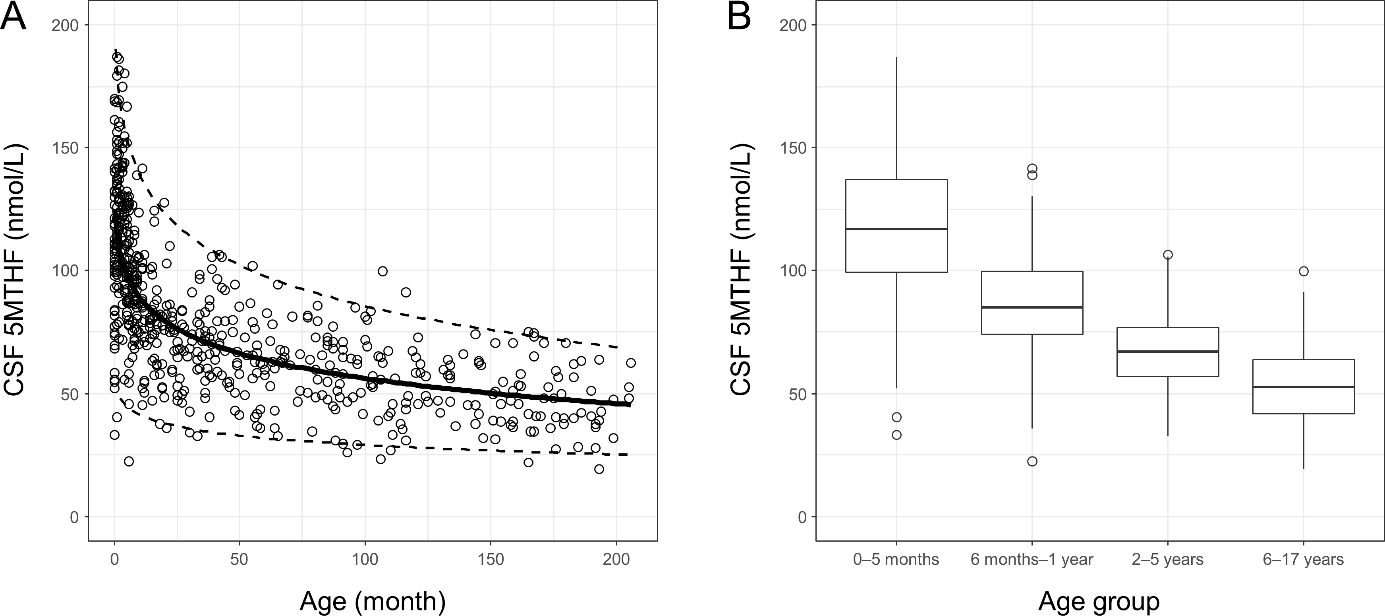


**Supplementary Figure 3**

Concentrations of 5MTHF and total folate in serum and CSF samples from pediatric neurological patients without folate supplementation or signs of peripheral folate deficiency

5MTHF, 5-methyltetrahydrofolic acid; CSF, cerebrospinal fluid


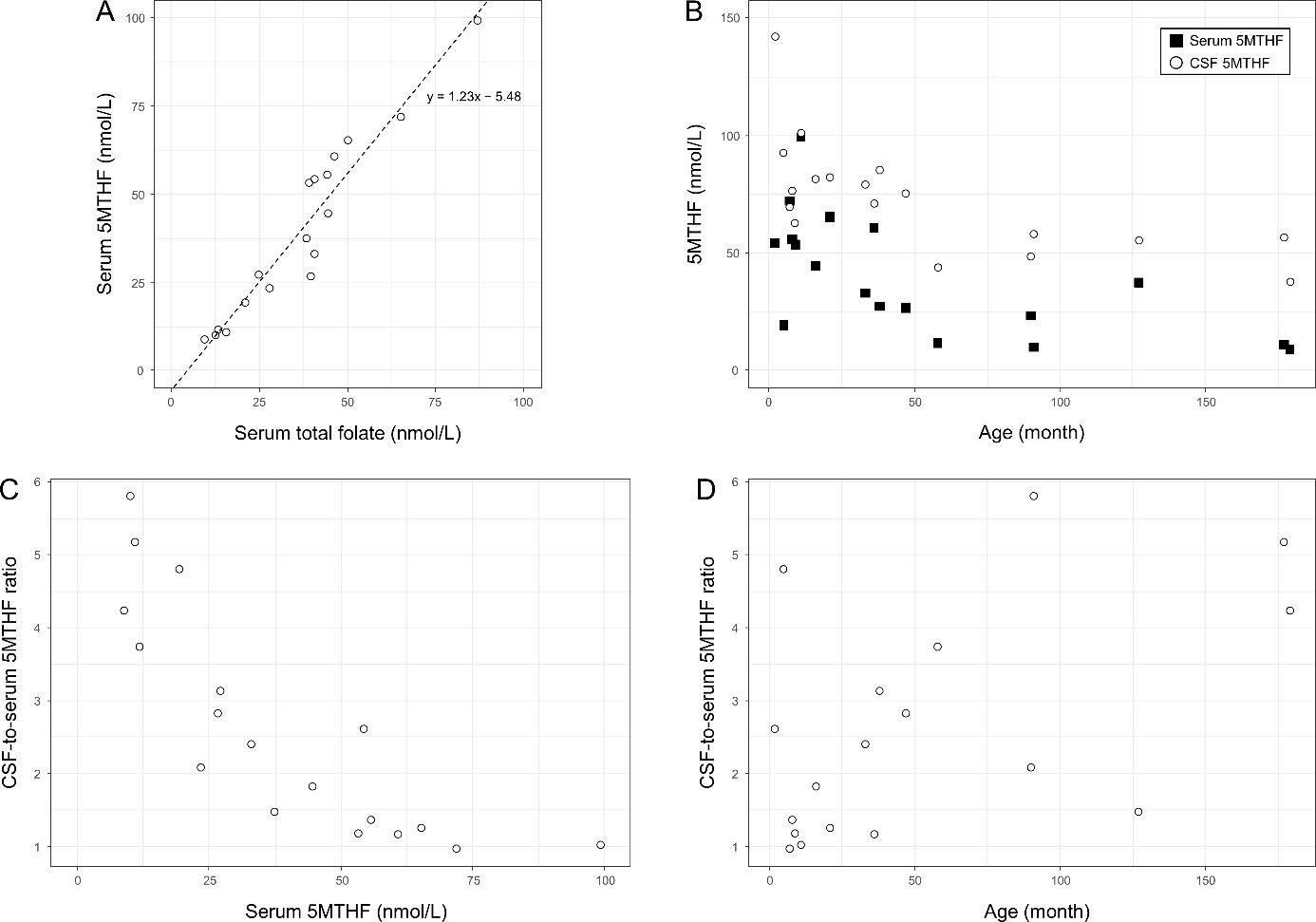


## Supplementary Table 1

Concentrations of 5MTHF and total folate in serum and CSF samples from pediatric neurological patients without folate supplementation or signs of peripheral folate deficiency

| Age | Plasma total Hcy (µmol/L) | Serum TF (nmol/L) | Serum 5MTHF (nmol/L) | CSF 5MTHF (nmol/L) | CSF-to-serum 5MTHF ratio | CSF 5MTHF-to- serum TF ratio | Serum 5MTHF − serum TF (nmol/L) | % difference |
| --- | --- | --- | --- | --- | --- | --- | --- | --- |
| 0y 2m | 9.6 | 40.6 | 54.3 | 141.9 | 2.6 | 3.5 | 13.7 | 33.7 |
| 0y 5m | 10.8 | 20.9 | 19.3 | 92.6 | 4.8 | 4.4 | −1.6 | −7.7 |
| 0y 7m | 9.0 | 65.2 | 72.0 | 69.6 | 1.0 | 1.1 | 6.8 | 10.4 |
| 0y 8m | 5.8 | 44.2 | 55.7 | 76.3 | 1.4 | 1.7 | 11.5 | 26.0 |
| 0y 9m | 5.2 | 39.2 | 53.4 | 62.8 | 1.2 | 1.6 | 14.2 | 36.2 |
| 0y 11m | 4.5 | 87.0 | 99.3 | 100.9 | 1.0 | 1.2 | 12.3 | 14.1 |
| 1y 4m | 4.9 | 44.6 | 44.6 | 81.3 | 1.8 | 1.8 | 0 | 0 |
| 1y 9m | 5.4 | 50.1 | 65.3 | 82.3 | 1.3 | 1.6 | 15.2 | 30.3 |
| 2y 9m | 6.4 | 40.6 | 33.0 | 79.2 | 2.4 | 2.0 | −7.6 | −18.7 |
| 3y 0m | 4.1 | 46.2 | 60.8 | 71.2 | 1.2 | 1.5 | 14.6 | 31.6 |
| 3y 2m | 6.8 | 24.7 | 27.2 | 85.2 | 3.1 | 3.4 | 2.5 | 10.1 |
| 3y 11m | 5.0 | 39.6 | 26.6 | 75.2 | 2.8 | 1.9 | −13.0 | −32.8 |
| 4y 10m | 10.3 | 13.3 | 11.7 | 43.7 | 3.7 | 3.3 | −1.6 | −12.0 |
| 7y 6m | 6.3 | 27.9 | 23.3 | 48.5 | 2.1 | 1.7 | −4.6 | −16.5 |
| 7y 7m | 8.1 | 12.6 | 10.0 | 58.0 | 5.8 | 4.6 | −2.6 | −20.6 |
| 10y 7m | 5.5 | 38.3 | 37.4 | 55.5 | 1.5 | 1.4 | −0.9 | −2.3 |
| 14y 9m | 10.3 | 15.7 | 10.9 | 56.4 | 5.2 | 3.6 | −4.8 | −30.6 |
| 14y 11m | 9.0 | 9.4 | 8.9 | 37.7 | 4.2 | 4.0 | −0.5 | −5.3 |

5MTHF, 5-methyltetrahydrofolic acid; CSF, cerebrospinal fluid; Hcy, homocysteine; m, months; TF, total folate; y, year

## Supplementary Table 2

Concentrations of 5MTHF and total folate in serum and CSF samples in the patient with Kearns-Sayre syndrome

| Age | Serum | |  | CSF | |  | CSF-to-serum 5MTHF ratio | Medications | |
| --- | --- | --- | --- | --- | --- | --- | --- | --- | --- |
|  | 5MTHF (nmol/L) | Total folate (nmol/L) |  | 5MTHF (nmol/L) | Total folate (nmol/L) |  |  | Folic acid (mg/day) | Folinic acid (mg/day) |
| 13y 0m |  | 16.0 |  | 3.9 | 3.9 |  |  |  |  |
| 17y 0m |  | 9062 |  | 22.7 | 159 |  |  | 20 |  |
| 18y 0m | 379 | 18577 |  | 23.4 | 149 |  | 0.06 | 20 | 25 |
| 18y 4m | 403 | 625 |  | 30.1 | 25.0 |  | 0.07 |  | 12.5 |
| 18y 11m | 255 | 258 |  | 40.0 | 31.0 |  | 0.16 |  | 25 |
| 19y 11m | 827 | 884 |  | 43.2 | 38.3 |  | 0.05 |  | 25 |

5MTHF, 5-methyltetrahydrofolic acid; CSF, cerebrospinal fluid; m, months; y, year

## Supplementary Table 3

Concentrations of 5MTHF and total folate in serum and CSF samples in the patient homozygous for *MTHFR* C677T polymorphism

| Age | Serum | |  | CSF | |  | CSF-to-serum 5MTHF ratio | Folic acid (mg/day) |
| --- | --- | --- | --- | --- | --- | --- | --- | --- |
|  | 5MTHF (nmol/L) | Total folate (nmol/L) |  | 5MTHF (nmol/L) | Total folate (nmol/L) |  |  |  |
| 65y 6m |  |  |  | 11.5 | 12.5 |  |  |  |
| 65y 9m | 4.0* |  |  | 12.2 |  |  | 3.05* |  |
| 65y 10m | 55.7 | 910.7 |  | 16.4 | 69.3 |  | 0.29 | 15 |
| 66y 0m | 32.6 | 51.4 |  | 27.7 | 28.1 |  | 0.85 | 0.7 |

* This serum sample was obtained on the day before lumbar puncture.

5MTHF, 5-methyltetrahydrofolic acid; CSF, cerebrospinal fluid; m, months; y, year
